# Parallelisms and deviations: two fundamentals of an aesthetics of poetic diction

**DOI:** 10.1098/rstb.2022.0424

**Published:** 2024-01-29

**Authors:** Winfried Menninghaus, Valentin Wagner, Ines Schindler, Christine A. Knoop, Stefan Blohm, Klaus Frieler, Mathias Scharinger

**Affiliations:** ^1^ Language and Literature, Max Planck Institute for Empirical Aesthetics, 60322 Frankfurt am Main, Hessen, Germany; ^2^ Faculty of Humanities and Social Sciences, Helmut-Schmidt-University/University of the Armed Forces Hamburg, 22043 Hamburg, Germany; ^3^ Seminar of Media Education, Europa-Universität Flensburg, 24943 Flensburg, Schleswig-Holstein, Germany; ^4^ Pragmatics, Leibniz Institute for the German Language, 68161 Mannheim, Baden-Württemberg, Germany; ^5^ Scientific Services, Max Planck Institute for Empirical Aesthetics, 60322 Frankfurt am Main, Hessen, Germany; ^6^ German Studies and Arts, Philipps-Universität Marburg, 35032 Marburg, Germany

**Keywords:** poetic language, aesthetics, parallelism, deviation, predictive coding

## Abstract

Poetic diction routinely involves two complementary classes of features: (i) parallelisms, i.e. repetitive patterns (rhyme, metre, alliteration, etc.) that enhance the predictability of upcoming words, and (ii) poetic deviations that challenge standard expectations/predictions regarding regular word form and order. The present study investigated how these two prediction-modulating fundamentals of poetic diction affect the cognitive processing and aesthetic evaluation of poems, humoristic couplets and proverbs. We developed quantitative measures of these two groups of text features. Across the three text genres, higher deviation scores reduced both comprehensibility and aesthetic liking whereas higher parallelism scores enhanced these. The positive effects of parallelism are significantly stronger than the concurrent negative effects of the features of deviation. These results are in accord with the hypothesis that art reception involves an interplay of prediction errors and prediction error minimization, with the latter paving the way for processing fluency and aesthetic liking.

This article is part of the theme issue ‘Art, aesthetics and predictive processing: theoretical and empirical perspectives’.

## 1. Introduction

Older theories of art reception already emphasized the importance of occasional disappointments or even violations of expectations [1]. Similarly, the ‘predictive processing’ hypothesis considers departures from expectations quite generally to be a crucial factor in many cognitive processes, including art reception. If recipients successfully cope with these challenges and manage to integrate the deviant phenomena into an updated mental model of the artwork, this may result in more comprehensive, more fluent and in the end also more enjoyable processing [2–4].

Verbal art is temporal, i.e. during incremental processing, recipients build up both syntax-, content- and literary style-based predictions regarding expectable continuations [5–7]. The present study investigates how the presence versus absence of parallelism and deviation––features that are both likely to modulate the predictability of the wording––affect the aesthetic evaluation of poems, humouristic couplets and proverbs. As an illustration of these two classes of features, consider the first line of Shakespeare's sonnet 34:

‘All days are nights to see till I see thee.’

### (a) Parallelisms

All words of this verse are monosyllabic and their rhythm conforms to an iambic metre. Moreover, the verse features a line-internal rhyme (‘see’–‘thee’). Following Jakobson [8,9] (see also [10,11]), we subsume all such features of (varied) repetition on all linguistic levels (phonology, morphology, syntax and semantics) under the concept of parallelism. Metre, rhyme and alliteration are only the most well-known pertinent features which, in the creative process, constrain the selection and combination of words (e.g. [12]). In processes of reading, systematic repetitions narrow the set of possible continuations and serve to build expectations/predictions regarding upcoming words [13–15].

Parallelistic patterning in language can be considered a temporal analogue to spatial symmetry and mirroring in visual aesthetics. Jakobson [8] emphasized that, beyond poetry, many commercial ads and political slogans also feature parallelistic patterns. The same has been shown for infant-directed speech (especially on the level of prosody; see [16,17]), natural conversation [18] and ritualistic speech, including prayers [11,19]. Parallelistic patterning is thus found across virtually all registers of language.

Crucially, parallelistic patterning seems to be a broadly applicable means of increasing both the aesthetic appeal of verbal utterances as well as the impact of the message they convey [20,21]. For instance, Menninghaus *et al.* [22] reported that metre and rhyme intensify impressions of sadness in response to sad poems and of joy in responses to joyful poems, along with enhancing ratings for beauty, liking and other aesthetically evaluative dimensions.

### (b) Deviations from canonical language use

In online processing, the first four words of Shakespeare's sonnet 34––‘all days are nights’––are likely to strike readers as semantically/logically paradoxical, regardless of the fact that this irritation will soon be resolved by the subsequent words ‘to see.’ Similar departures from predictable wording, such as incomplete word forms and non-canonical grammar, frequently co-occur with parallelisms or even serve to implement them (cf. [23,24]). Contrary to outright ‘vices’ of poetic diction, such acceptably deviant features are considered ‘poetically licensed’ (cf. [25,26]) and may enhance recipients' subjective impressions of genre-specific aesthetic virtues [27].

Thierry *et al.* [28] investigated how a strong type of deviation, namely, noun-to-verb conversions––for instance, (to) ‘lip something’ instead of (to) ‘speak’ or ‘whisper something’––affects the neurophysiology of sentence processing. Compared to conventional versions of sentences with a convergent meaning, the original deviant wording elicited significant modulations of the event-related potentials derived from the human scalp by means of electroencephalography (EEG)––specifically, modulations of the left-anterior negativity (LAN) and the P600 (a positivity occurring 600 ms post stimulus onset)––, but not the stronger N400 responses (a negativity at 400 ms post stimulus onset) that are held to be indicative of higher cognitive processing demand. Apparently, because lip movements are part of the physiology of speaking, semantic understanding is straightforward enough, regardless of the grammatical irritation. However, subjective comprehensibility ratings and potential positive or negative aesthetic effects of noun-to-verb conversions were not targeted in this study.

Blohm *et al.* [27] investigated short single sentences extracted from German poems along with author-created sentence variants thereof for the effects of canonical versus deviant syntax and/or morphology. Deviant variants were rated as sounding less ‘natural,’ yet also more ‘poetic.’ The co-presence of a sustained alternating stress pattern throughout the sentences––and hence of a feature of ongoing rhythmic/metrical parallelism––increased this effect.

### (c) The present study

Empirical research into music perception has shown that quantifiable formal properties such as rhythmic and melodic features lead recipients to build up expectations during listening, and that these are crucial to subjective aesthetic experience [29,30]. Moreover, the recurrent melodic properties of poem recitations––as objectively measured by acoustic autocorrelation analyses––enhance the perceived musical and aesthetic qualities of recited poems and their song versions [31,32].

Continuing this line of research, we developed novel quantitative measures that capture the frequency/density of patterns of parallelism and deviation based on analyses of formal linguistic text features. Having calculated these measures for the poems, proverbs and humoristic couplets presented in our study, we used the resulting scores as predictors of readers' cognitive and aesthetic judgements of these texts. This approach has substantial potential to shed new light on how the two prediction-modulating fundamentals of poetic diction, i.e. parallelism and deviations, affect cognitive processing and aesthetic evaluations of texts written in verse via processes of prediction and prediction error minimization.

Patterns of deviation typically occur less frequently and less predictably than patterns of parallelism. However, since they often serve the very implementation of ongoing metre and hence of prosodic parallelism, we did not take it for granted that deviant features necessarily reduce subjective impressions of processing ease and aesthetically relevant qualities. Rather, we also tested three hypotheses that assign these features potentially inherent positive contributions to aesthetic perception.

### (d) The hypotheses investigated in the present study

Varied repetition is the essence of parallelistic patterning not only, but most notably in verse [33]. The repetitive nature of parallelism gives rise to linguistic predictions (e.g. [34]) and hence should enhance the processing of the stream of words read, thus resulting in higher processing fluency. By contrast, deviations from standard linguistic expectations should result in less accurate linguistic predictions, thereby rendering the processing less fluent. The standard hypothesis of fluency-driven aesthetic liking [35,36], therefore, predicts positive effects of parallelisms (= Hypothesis 1) and negative effects of deviation features on cognitive and aesthetic processing (= Hypothesis 2).

We tested these complementary hypotheses regarding the nexus between fluency and aesthetic liking as separate Hypotheses 1 and 2, because there is dissent regarding the hypothetically merely negative effects of disfluency. For instance, the pleasure-interest model of aesthetic liking [37,38] maintains that cognitively challenging stimulus features may arouse interest and enhance aesthetic liking if recipients' attempts to cope with these features are successful. We tested this hypothesis as Hypothesis 2a.

In a similar vein, we expected that subjectively perceived ‘praegnanz’ [39] might be enhanced not only by features of parallelism, but also by features of deviation (= Hypothesis 2b). The underlying reasoning was that omitting words or parts thereof may render the resulting wording unusually compact and more salient and in this sense higher in ‘praegnanz.’ In the verbal domain, the concept of praegnanz might be used to designate higher order (holistic) properties of verbal expressions that are (i) perceptually striking and of an least apparent simplicity regarding their inner organization, (ii) highly effective in communicating their meaning and, as a consequence, also (iii) highly memorable. Menninghaus *et al*. [40] reported that in the assessment of short sentences, specifically proverbs, praegnanz ratings capture an important aesthetic dimension and that German-speaking participants readily have an intuitive understanding of the task to rate a proverb for praegnanz.

Regarding the rating variable ‘poetic’ (German ‘poetisch’, cf. a classical treatise on what makes poems ‘poetic‘ [41] and a more recent account [42]), we expected that an intuitive understanding of this adjective in the context of a study on poems, humouristic verses and proverbs should include an overall positive evaluation of the virtues of ‘poetic’ diction, i.e. one that extends beyond the features of parallelism to those of deviation (= Hypothesis 2c; for similar hypotheses of positive effects of prediction errors on aesthetic appreciation, see [3,43]).

Theoretically, texts that push parallelistic patterning to extreme levels would consist in serial repetitions of the very same words or phrases. In this regard, Stein's [44, p. 178] ‘A rose is a rose is a rose is a rose…’ could be understood as testing the limit at which the message-reinforcing power of parallelism turns into monotony and even tautology. One might, therefore, suspect that the positive effect of parallelistic patterning on aesthetic liking might decrease or even reverse after a certain point. Regarding the opposite––i.e. pushing complexity rather than seriality/monotony to ever higher levels––Berlyne [45] has observed that there might be an inverted U-shaped relationship between complexity and liking: as complexity increases so does liking, but only up to an optimal point, after which liking decreases as complexity keeps increasing (for recent empirical evidence in the context of predictive processing and music, see [46]). We, therefore, tested whether we would find indications of a curvilinear relationship between text-based parallelism scores and aesthetic evaluations already in the fairly prototypical poems we presented in this study (= Hypothesis 3).

Different genres of literature are likely to be associated with different types of challenges and rewards for readers. Under this assumption, we tested all participants for a preference for poetry and for rhythmic ability (for details, see the Methods section below). We did so with the expectation that, in the case of participants with a high preference for poetry and high rhythmic ability, the text scores for parallelism should be stronger predictors of the aesthetically evaluative ratings than for participants who were low in poetry preference and rhythmic ability (= Hypothesis 4).

## 2. Methods

### (a) Participants

Participants were recruited in two steps through the Prolific participant pool (www.prolific.co). First, we conducted an online screening study (*n* = 532; for details, see [47]), in which we collected demographic data and person-related information regarding (i) literature-related preferences and poetry preference specifically (how much participants like to read or listen to poems indicated on a 7-point scale ranging from 1 = *not at all* up to 7 = *very much*), reading and writing frequencies, including the estimated number of hours per week spent on reading poems during the time of their lives when their literary interest was greatest, (ii) musical preferences and abilities, especially the musical rhythm ability test (RAT) [48], and (iii) other personality characteristics such as aesthetic responsiveness, Big Five personality traits and general intelligence, that were not analysed in this article.

Participants who expressed interest in participating in the actual study (*n* = 437) were assigned to one of six groups based on their poetry preference and engagement with poetry reading and writing (low, medium, high) and their performance in the RAT (low, high). Specifically, we formed three groups distinguishing participants who do not like poems (low; liking ratings of 1 or 2 and less than one hour of weekly poetry reading), who reported an average liking of poetry (medium; liking ratings of 3–5), and who like poetry a lot (high; liking ratings of 6 or 7 and at least one hour of weekly poetry reading). Each of the three groups was then split into participants with low scores and high scores on the RAT. Since we aimed for subsamples of 40 participants per group, i.e. 240 participants for the main study, we oversampled and invited 304 persons to participate in the main study. Participants gave their informed consent by explicitly stating their agreement. All procedures of the study were ethically approved by the Ethics Committee of the Max Planck Society (no. 2017_12) and were undertaken with informed consent of each participant.

The final sample included 282 participants (159 female, 117 male, six other or undisclosed) with a mean age of 33.1 years (s.d. = 11.2, median 30, range 18–71). Even though the sample size in the screening study was twice as large as we had aimed for, we were unable to fill the groups evenly. For the low RAT group, the grouping dependent on low, medium and high poetry preferences resulted in 11, 57 and 20 participants per group. For the high RAT group, the corresponding figures were 55, 105 and 34 participants ($\chi_{2}^{2}=8.55$, *p* = .014).

The mean poetry preference rating was 3.88 (s.d. = 1.78, median = 4) with nearly no skew (0.03) and a negative kurtosis (−1.07). The mean RAT score was 0.25 (s.d. = 0.87, median = 0.39) with a negative skew (−1.07) and a positive kurtosis (1.10) (see the electronic supplementary material, figure S1).

### (b) Stimuli

We used three sets of texts––proverbs [40], humouristic couplets [49] and poems [22]––that were previously used to test rhyme and metre effects on subjective aesthetic evaluations, although without quantitative measures of the features of parallelism and deviation. To control for effects of familiarity [50,51], all texts used in these studies were pre-tested for familiarity in comparable samples of participants.

In order to disentangle the effects of the parallelistic target features metre and rhyme from the content-based variance of the texts, the three studies presented their respective texts in (i) the original and three systematically modified versions: (ii) metre, but not rhyme, (iii) rhyme, but not metre, and (iv) neither rhyme nor metre.

The modified versions of the shortest text genre (proverbs) are typically one or two words longer than the original versions. This was an explicit strategy, as the rhetorical/poetic quality of proverbs hinges specifically on making their messages as short, compact and succinct as possible. Experimentally modifying brevitas-driven ‘praegnanz’, therefore, almost mandates a few more syllables/words. As shown and discussed in Menninghaus *et al*. [40], these extra syllables render the wording perceptually less succinct and memorable, yet also easier to process conceptually (for the distinction between perceptual and conceptual fluency, see [52] and [36]). For similar reasons, the experimental modifications in the reference studies on full-length poems [22] and humouristic couplets [49] were performed with a license for a few extra syllables. However, this license was applied with so much restraint that the total number of words and syllables does not differ significantly between versions.

Reuse of the previously built corpora was not solely motivated by convenience; it also allowed us to test for the replicability of our findings. However, using these text corpora also involved a limitation: whereas the parallelistic target features metre and rhyme were varied independently in these corpora, the deviation features were not varied independently of the rhyme and metre manipulations. In fact, ‘normalizing’ the deviation features was frequently one of the means for also deactivating rhyme and/or metre. This is legitimate as deviation features are often functional for the very implementation of high levels of parallelism in the first place. As a result, poem versions low in features of parallelism, yet high in features of deviation were not part of the reference studies. As such poems are barely found in the more generally known poetic tradition, they are likely to elicit a negative prototypicality effect. Still, the absence of such versions is a limitation of our experimental design. Specifically, the results for the deviation features can, therefore, only be a first step and call for further testing in other experimental designs that fully dissociate the variables of parallelism and deviation.

The three previous studies involved 60 humouristic couplets, 40 poems and 32 proverbs, respectively. In order to keep the number of texts per genre balanced, we chose 32 items each out of the 60 humouristic couplets and the 40 poems. In this process, we took care to select items with the largest difference in the total parallelism score (see below under *Text measures*) between their original and no rhyme/no metre versions. In some cases, we slightly revised the wording of the modified text versions, especially in the case of the proverbs, in order to ensure a more systematic manipulation of rhyme and metre throughout the three text corpora.

The meticulous linguistic analyses of the features of parallelism and deviation across all four versions of the texts and the resulting scores are reported in great detail in an open access repository that also includes all German texts presented (https://osf.io/dcre9/). Here, we only provide a short example of our experimental modifications by referring again to the initial line from Shakespeare's sonnet 34:

(i) ‘All days are nights to see, till I see thee.’

Modifications of the types described above yield the following experimental sentence versions (for an analogous experimental modification of features of parallelism performed on the first stanza of William Blake's ‘Ah Sun-flower!’, see [53]):

(i) without the line-internal rhyme: ‘All days are nights to see, till I see *you.*’;
(ii) without ongoing iambic metre: ‘All days are nights to see, *un*til I see thee.’; and
(iii) without both ongoing metre and internal rhyme: ‘All days are nights to see, *un*til I see *you*.’.

Regarding the deviant, because temporarily paradoxical wording, ‘All days are nights,’ a more canonical variant––such as ‘All days *appear* (or *feel*) *like* nights […]’––allows us to test for the cognitive and aesthetic effects of this temporary deviation from standard expectations/predictions.

### (c) Design

The online experiment was set up in Labvanced (www.labvanced.com). It included four sessions per participant that were conducted with at least one day between subsequent sessions. Text genres (Poems, Humoristic Couplets, Proverbs) were presented session-wise within-participant. One session contained all proverbs and another one all humoristic couplets. Because the 32 poems take far longer to read than proverbs or couplets, they were presented in two separate sessions, one including all joyfully moving poems and one all sadly moving poems. Using a Latin Square design, we identified four session orders and distributed the participants in the six groups about equally across the four orders. Each participant was presented with all three text genres, reading each of the 96 texts in one of its four versions (original, no rhyme, no metre, no rhyme and no metre). The text order within each session was randomized for each participant. A session lasted between half an hour and an hour.

### (d) Text measures

Rhyme and metre stand out as being typically the only ongoing variants of parallelism in poetic diction. Still, they by no means account for all parallelistic features of texts. The experimentally de-metred and/or de-rhymed poem variants presented in our study all retain several more local parallelistic features; even free verse poems routinely include local features of parallelism.

Our newly developed measures are designed to reflect the average number of phonological, morphological, syntactic and semantic features of parallelism, respectively, as computed per syllable of a given text. We additionally coded scores for parallelistic patterns in the alignment of verse units and syntactic units. Adding these five scores yielded an additional score that reflects the average total number of parallelistic patterns per syllable of a given text. Analogously, we computed the average number of phonological, morphological, syntactic and lexical deviations per syllable as well as the average total number of deviations. We henceforth refer to the resulting total *scores* for the features of parallelism and deviation as Parallelism and Deviation. By contrast, references to the features themselves are not capitalized. For all details regarding the measures applied and the scores obtained for the texts, see https://osf.io/dcre9/.

For the purposes of the present study, we exclusively drew on the total scores for the two groups of text features. Table 1 shows the mean, minimum and maximum scores of Parallelism and Deviation for the four versions of the three text corpora used in the present study (see also the electronic supplementary material, figure S2):

**Table 1.** Parallelism and deviation scores.

	poems		humouristic couplets		proverbs	
	mean	(min–max)	mean	(min–max)	mean	(min–max)
parallelism						
original	5.92	(4.45–7.75)	4.49	(2.78–7.50)	5.32	(2.14–8.14)
no rhyme	4.67	(3.34–6.66)	3.22	(1.56–6.50)	3.14	(0.88–6.13)
no metre	4.08	(2.98–5.59)	3.23	(1.59–5.20)	2.38	(0.90–4.50)
no rhyme and no metre	3.22	(2.17–4.75)	2.21	(0.53–4.53)	0.98	(0.00–3.00)
deviation						
original	0.250	(0.158–0.440)	0.149	(0.000–0.562)	0.342	(0.000–1.000)
no rhyme	0.263	(0.141–0.495)	0.158	(0.000–0.375)	0.260	(0.000–0.750)
no metre	0.156	(0.039–0.270)	0.129	(0.000–0.500)	0.144	(0.000–0.500)
no rhyme and no metre	0.128	(0.019–0.225)	0.136	(0.000–0.500)	0.033	(0.000–0.300)

Across the three text genres, each individual syllable is on average part of more than four different parallelistic patterns, with the longest text genre (poems) scoring highest. The total deviation scores per syllable are at a far lower absolute level, with the shortest text genre (proverbs) scoring highest.

### (e) Measures of subjective perception (ratings)

We assessed (i) cognitive processing dimensions that we expected to be relevant for all three text genres, (ii) general aesthetically evaluative dimensions, and (iii) music-analogous dimensions of poetic diction. In addition, we collected (iv) some ratings that were selected with a special focus on only one of the text genres.

Ease of cognitive processing was assessed by participants' retrospective ratings for the degree to which they perceived the texts as ‘comprehensible,’ with ratings for ‘confusing’ as a reverse analogon.

The more general aesthetically evaluative rating dimensions included ‘beautiful’ and ‘liked’ as well as ‘intuitively accessible’ [German ‘anschaulich’] and ‘vivid’ [German ‘lebendig’]. ‘Vivid’ and ‘beautiful’ ratings have explained relevant variance in the liking of poems in earlier empirical studies ([22,54]; for the theoretical tradition regarding aesthetic ‘vividness,’ cf. [55]).

The items ‘rhythmic,’ ‘melodious’ and ‘poetic’ were chosen because they were among the most frequently listed adjectives in a study that explored the verbal concepts that non-expert readers associate with poetry [42] and were also predictors of aesthetic liking in earlier studies on poetry [31,32].

Ratings for ‘surprising’ and ‘witty’ were included with special reference to the humouristic couplets. Given that definitions of humour emphasize the importance of surprising turns in trains of thought [56,57], humouristic couplets should score relatively higher than prototypical poems and proverbs do on ratings for ‘witty’ and ‘surprising.’

The rating item ‘moving’ was chosen because it has a long tradition in poetics since Latin antiquity and has already been shown to capture an important emotional dimension specifically in responses to the selected poems [22,58].

For the reasons why we included the rating items ‘interesting,’ ‘poetic’ and ‘praegnanz,’ see Hypotheses 2a–c in the Hypotheses section.

The majority of the ratings were given in response to questions of the type ‘How [….] do you find this poem/proverb/humoristic couplet?’ The liking and the familiarity ratings were asked for in a slightly different wording: ‘How much do you like this text?’ and ‘How familiar was this text for you prior to this study?’ All ratings were given on scales ranging from 1 = *not at all* to 7 = *very much* (*very familiar*, respectively).

### (f) Data preparation and statistical analysis

Regarding (i) cognitive processing, we calculated mean composite scores for the variable pairs ‘comprehensible’ and ‘confusing’ (reversed) [*Comprehensibility*]. Regarding (ii) general aesthetic evaluation, we calculated mean composite scores for ‘beautiful’ and ‘liked’ [*Beauty/Liking*] as well as for ‘vivid’ and ‘intuitively accessible’ [*Vividness*]. Regarding (iii) music-analogous aspects of poetic diction, we calculated mean scores for ‘melodious’ and ‘rhythmic’ [*Melodiousness*]. Variables were paired based on theoretical reasons and the empirical results of exploratory factor and cluster analyses (see the electronic supplementary material, text S1). (iv) We also separately analysed the ratings for ‘Interesting,’ ‘Poetic’ and ‘Praegnanz.’ (All rating variables used in the analysis––whether based on individual ratings or reflecting composite scores––are henceforth referred to in capital letters.)

The data have a counterbalanced cross-classified structure [59]: each text rating is clustered within 282 individuals and 96 texts. As we included in the analysis participants who did not complete all four sessions, our dataset comprises 25 261 ratings instead of 27 072, as would be expected without missing data. The dependent rating variables have been recoded to a range of 0–6 (instead of 1–7).

Since all experimental manipulations consisted in systematically removing the features of sustained parallelism (rhyme and metre), along with removing all or some of the more local features of deviation, the two text-based scores could be expected to show a more or less consistent downward trend across the four text versions of the three text genres and thus to correlate positively. We calculated the correlations between the parallelism and deviation scores across all text genres as well as separately for each text genre.

In order to test Hypotheses H1 and H2, H2a, b and c as well as H4, we conducted seven cross-classified multilevel analyses for the (derived) rating variables Melodiousness, Comprehensibility, Vividness, Beauty/Liking, as well as Praegnanz, Interesting and Poetic (for the R-code, see the electronic supplementary material, text S2). These analyses tested the combined effects of Parallelism and Deviation on the ratings given and how both the Text genre and the two person variables (Poetry Preference ratings and performance on the RAT) moderated these ratings. We modelled random intercepts and random slopes of the Parallelism and Deviation scores for both participants and texts as well as random slopes of Poetry Preference and RAT for texts. All metric predictor variables were *z*-standardized (*M* = 0 and s.d. = 1). The Text Genre variable was contrast-coded with Poems as the reference category.

We then tested the relationships between the Parallelism and Deviation scores and the aesthetically evaluative ratings for curvilinearity (=Hypothesis H3).

Finally, we examined how convergent the total effects of the Parallelism and Deviation scores are with those of the experimental manipulation of rhyme and metre (contrast-coded; for rhyme/metre = 1 and for no rhyme/no metre = −1, respectively). Rhyme and metre are the only features of parallelism that are observed throughout all individual lines and stanzas of many poems. The other patterns of parallelism typically show a far more local and far less frequent distribution across texts. For this reason, rhyme and metre were likely to account for the largest share of the Parallelism score. Still, because all the texts presented also include more local features of parallelism, the Parallelism score should explain some additional variance beyond that for metre and rhyme.

All analyses were conducted in the statistical environment R [60], using the *psych* [61] and *afex* [62] packages for the analyses and *ggeffects* [63], *emmeans* [64] and *ggplot2* [65] for the figures.

## 3. Results

### (a) Correlation between Parallelism and Deviation scores

The correlation between the text-based scores for Parallelism and Deviation is positive and significant if computed across all text genres and text versions (*r* = 0.40, *n* = 384, *p* < 0.001). The correlation is also positive and significant if computed separately for each text genre, with Poems yielding the highest correlation (*r* = 0.53, *n* = 128, *p* < 0.001) and Humouristic Couplets the lowest (*r* = 0.20, *n* = 128, *p* = 0.028). This result was expectable given that the original texts were invariably highest in both Parallelism and Deviation (see above under ‘Stimuli’). At the time, within the individual versions of each of the three text genres, no correlation is significant: they are lower in strength, and some are even negative. (For all details, see the electronic supplementary material, table S1.)

### (b) Effects of Parallelisms and Deviations on cognitive and aesthetically evaluative ratings

For all dependent variables, the Parallelism score predicted significant positive effects and the Deviation score significant negative effects (table 2; electronic supplementary material, tables S2 and S3). Thus, Hypotheses 1 and 2 were fully confirmed. By contrast, the alternative Hypotheses 2a, 2b and 2c, which stipulated a potential positive effect of the Deviation scores on select ratings (Interesting, Praegnanz, Poetic) were not confirmed. To be sure, we did find positive effects of the Deviation scores for some rating variables when Deviation was modelled separately as a single predictor. However, when the two text-based predictors (Parallelism and Deviation) were modelled conjointly, the Deviation score predicted only negative effects, while the positive effects of the Parallelism score increased.

**Table 2.** Coefficients of parallelism and deviation scores for the ratings. (Coefficients of parallelism and deviation with standard errors (s.e.) for the seven dependent variables (DVs); LL = lower limit and UL = upper limit of the 95% confidence interval (CI).)

rating (DV)	text score (predictor)	coefficient	s.e.	95% CI		*p*-value
				LL	UL	
beauty/liking	parallelism	0.52	0.034	0.46	0.59	<0.001
	deviation	−0.38	0.053	−0.49	−0.28	<0.001
melodiousness	parallelism	1.29	0.085	1.12	1.45	<0.001
	deviation	−0.90	0.203	−1.29	−0.50	<0.001
vividness	parallelism	0.44	0.032	0.37	0.50	<0.001
	deviation	−0.34	0.049	−0.43	−0.24	<0.001
comprehensibility	parallelism	0.31	0.036	0.24	0.38	<0.001
	deviation	−0.31	0.047	−0.40	−0.21	<0.001
poetic	parallelism	0.44	0.031	0.38	0.50	<0.001
	deviation	−0.22	0.045	−0.31	−0.13	<0.001
interesting	parallelism	0.32	0.026	0.26	0.37	<0.001
	deviation	−0.20	0.032	−0.27	−0.14	<0.001
praegnanz	parallelism	0.42	0.032	0.35	0.48	<0.001
	deviation	−0.22	0.044	−0.31	−0.13	<0.001

This result is indicative of a statistical suppression effect between two co-occurrent variables (see [66]; for details see the electronic supplementary material, text S3). Suppression effects are likely to occur when two predictor variables are correlated and account for shared variance in the stimuli. This is likely to be the case in our study, as all deviations found in rhymed and metred texts are invariably in accord with the requirements of rhyme and metre. In this regard, deviations are hence not opposites of the features of parallelism. In such cases, a joint as compared to a separate modelling of Parallelism and Deviation is likely to reduce the positive effects of Deviation and enhance those of Parallelism. This is exactly what the two analyses show in the present case.

The effects of the total Parallelism and Deviation scores on the rating variables were greatest for Humouristic Couplets and weakest for Proverbs. All interactions between Parallelism/Deviation scores and Text Genre are significant except for the interaction of Deviation and Text Genre for Melodiousness. The mean ratings per Text Genre are, for all but one rating variable, highest for Poems, followed by Humouristic Couplets and Proverbs. Comprehensibility is the exception: for this variable, Humouristic Couplets had the highest and Poems the lowest mean (for details see the electronic supplementary material, table S2).

### (c) Curvilinear effects of Parallelism and Deviation

**Figure 1.**
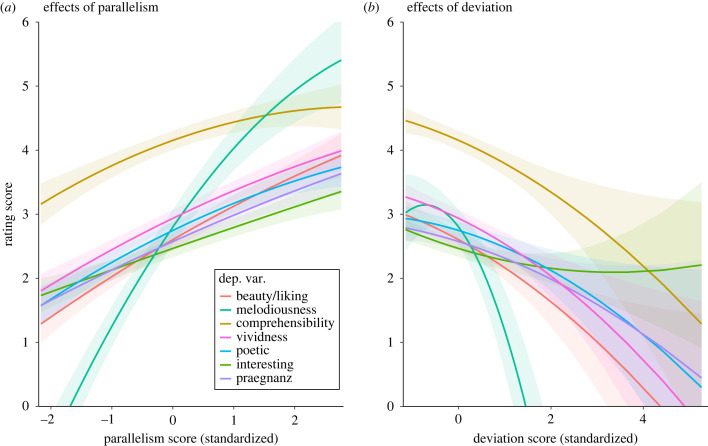
Estimated relationships between parallelism score (*a*) and deviation score (*b*) and the rating variables. Shaded areas depict the 95% CI band.

Supporting Hypothesis 3, analyses testing for a curvilinear relation between Parallelism and the aesthetically evaluative ratings by including a quadratic Parallelism term yielded significant effects for Melodiousness, Comprehensibility, Vividness and Poetic (figure 1; electronic supplementary material, tables S4 and S5). Regarding Melodiousness, the curvilinear effect was significantly stronger for Poems than for Proverbs and Humouristic Couplets. For the Deviation score, the analyses yielded only for Melodiousness a significant curvilinear effect. In addition, the results for Praegnanz show significant interactions of the quadratic term of the Deviation score with both Poetry Preference and RAT score.

### Effects of the person variables Poetry Preference and Rhythmic Ability

Supporting Hypothesis 4, Poetry Preference had a significant effect on all rating variables. Participants with a higher Poetry Preference rated the texts higher on all rating variables (for details see the electronic supplementary material, table S3). The largest effects of Poetry Preference were obtained for poems. Furthermore, higher Poetry Preference increased the positive effect of Parallelism on Melodiousness across all text genres. By contrast, for Beauty/Liking and Interesting, the moderating effect of Poetry Preference on the relationship between Parallelism and the rating variables was moderated by Text Genre. For Vividness, a higher Poetry Preference increased the negative effect of Deviations.

For Rhythmic Ability, the main effects and interactions were, with two exceptions, not significant. For Melodiousness, the effect of Rhythmic Ability was moderated by Text Genre, with participants scoring high on Rhythmic Ability providing higher ratings for Proverbs and Humouristic Couplets, but lower ratings for Poems. Furthermore, Participants scoring high on Rhythmic Ability had a stronger association between the Parallelism score and Melodiousness. Higher scores on Rhythmic Ability were also associated with higher ratings for Comprehensibility; this effect was largest for Humouristic Couplets and smallest for Poems.

Finally, for participants who were low in Rhythmic Ability, the negative effects of the Deviation scores on Comprehensibility were particularly strong. Or, from an inverse perspective, higher Rhythmic Ability is associated not only with a higher sensitivity to and competence regarding the features of parallelism, but also with a higher capability to integrate the deviation-driven prediction errors into an overall pleasurable trajectory.

### (d) Effects beyond rhyme and metre

When including the experimental manipulation of rhyme and metre in the statistical analyses, the Parallelism scores still had significant positive effects on the cognitive and aesthetically evaluative ratings except for Poetic and Praegnanz. By contrast, the effects of the Deviation scores were fully accounted for by the experimental manipulation and not significant anymore. The rhyme manipulation had for all ratings the strongest and most significant positive effects, in line with earlier findings that contemporary readers of German poems harbour strong expectations/predictions that poems be rhymed [67]. Metre had no significant effects on the ratings except for Comprehensibility. The interaction of rhyme and metre had small significant effects for the variables Melodiousness, Interesting and Praegnanz. For all details see the electronic supplementary material, tables S6 and S7.

## 4. Summary and discussion

Sustained parallelistic patterns, such as metre and rhyme, exert a particularly strong constraint on the selection and combination of words, thereby rendering the wording of upcoming sentences more predictable. By contrast, the concurrent features of deviation compromise canonical expectations/predictions regarding correct word form and word order. The quantification of parallelisms and deviations as proposed in our study, therefore, opens up opportunities to investigate poetic diction within the Predictive Processing framework. The scores for Deviations versus Parallelisms show across all three text genres a substantial power to predict negative versus positive cognitive and aesthetic effects. If, as it is reasonable to assume, parallelism and deviation influence our predictive processes during reading (i.e. enhance or reduce the predictability of the verbal chain), these results offer important directions for a predictive processing approach to the aesthetic appreciation of poetic texts.

Notably, this result is of a previously not observed type insofar as the complementary findings were not made for different stimuli, but for different dimensions of the very same texts, and, moreover, not for single features of parallelism and deviation, but for the combined effect of a great number of concurrent individual features from both categories as reflected by our two new measures. Furthermore, the analyses regarding the explanatory power of the Parallelism and Deviation scores beyond the rhyme and metre manipulation show that these fine-grained measures—especially the Parallelism score—account for variance not explained by rhyme and metre, but by other, more local features of parallelism. These text scores can be particularly useful in studies that address parallelism in all sorts of prose texts.

None of the three hypotheses that predicted positive contributions of the deviations to aesthetic liking via higher ratings for ‘interesting,’ ‘praegnanz’ and ‘poetic’ (Hypotheses 2a, 2b and 2c) was confirmed, at least not when the variables Parallelism and Deviation were modelled as joint predictors of the ratings. Our study design hence did not yield evidence for a direct positive effect of Deviation on the aesthetic evaluations. Still, the fact that the original text versions which received the highest aesthetic evaluations are not only highest in Parallelism, but also in Deviation, clearly supports the assumption that the features of deviation are at least compatible with and do not counteract the positive effects of parallelism. In this interpretation, our results are well compatible with the predictive processing hypothesis about aesthetic liking. After all, according to this hypothesis, aesthetic liking does not arise from experiencing prediction errors (i.e. deviations) *per se*, but from successfully coping specifically with prediction errors driven by linguistic deviations that are combined with parallelistic patterns.

The contrast to Graf & Landwehr's [38] findings that some visual design features which require extra cognitive efforts support positive ‘interest’ effects may be explained by the difference between the aesthetic domains (spatial/visual versus temporal/language-based aesthetics). The absence of a positive effect of deviation on the ‘poetic’ ratings as reported by Blohm *et al*. [27] can be attributed to the fact that, in this previous study, ‘poetic’ was the only rating item besides ‘natural.’ In this context, the word ‘poetic’ might have been primarily understood as a broad counterpart to a more ‘natural’ diction. By contrast, the semantic environment was very different for the raters in the present study: they had to make subtle distinctions between ratings for ‘poetic’ and those for ‘beautiful’, ‘vivid,’ ‘praegnanz,’ ‘interesting,’ etc. Moreover, the studies by Thierry *et al*. [28] and Blohm *et al*. [27] targeted only one carefully controlled deviation within the confines of short single sentences, whereas our stimuli mostly included several features of deviation and were partly full-length poems.

Our finding that the Parallelism score is a particularly strong predictor of Melodiousness lends an additional, novel type of support to the results of earlier studies on the topical poem–song affinity [32].

Our study also provides, to our knowledge, the first evidence that, as Parallelism scores increase, the strength of the positive effect of Parallelism on aesthetic judgements decreases. Only for Comprehensibility did we find a plateau effect, i.e. further increases in parallelism no longer have any additional positive effect on the ratings, but also not a negative effect. This, too, is consistent with the predictive processing theory: stimuli that are too predictable do not afford room for prediction error minimization to a significant degree and may, therefore, not support higher levels of aesthetic liking. Regarding Deviation, we observed for most rating variables no such nonlinear effects; rather, the negative effects increase as the level of Deviation increases. Only for Melodiousness did the negative effect of Deviation even get significantly stronger with increasing levels of Deviation. Importantly, as we noted above, the opposite effects of Parallelism and Deviation are not mutually exclusive, as both effects are strongest for the very same text versions, namely, the original versions which feature across all text genres the highest levels of both Parallelism and Deviation.

Finally, comparing poetry with the more mundane genres of proverbs and humouristic couplets yielded an interesting pattern: Poems are higher than the two other genres in Parallelism scores and aesthetically evaluative ratings, but at the same time, they are lowest in Comprehensibility. This finding is not readily compatible with the standard cognitive fluency hypothesis of aesthetic liking. It can be interpreted as highlighting the importance of going through relatively high levels of prediction errors (that is, cognitive challenges) in order to reach those higher levels of processing reward that are associated with the most complex text genre examined in our study, that is, poetry (cf. [3]).

### (a) Limitations

The two groups of features that are the object of the present study (parallelisms and deviations) by no means cover all aspects of poetic diction. Specifically, metaphors, similes, allegories and other devices of poetic imagery lie beyond the scope of the present study. Once again taking Shakespeare's line ‘All days are nights to see till I see thee’ as an example, this line is also readily readable as a semantic figure, specifically, as a hyperbolic image of outstanding beauty. A more comprehensive account of poetic diction should, therefore, also include the various forms of figurative meanings and poetic imagery.

### (b) Future directions

In the study reported in this paper, we exclusively drew on unfamiliar texts from three text genres. However, an earlier study that was wholly devoted to proverbs [68] compared the effects of single features of deviations on cognitive and aesthetic processing for an equal number of familiar and unfamiliar proverbs. Combining eye tracking with subjective ratings, the study reported adverse effects of the deviation feature on cognitive processing and aesthetics evaluations for the unfamiliar, but not for the familiar proverbs. This result is meaningful form a predictive processing perspective, as familiarity––being the result of experience-shaped priors—enhances the predictability of upcoming words. It is hence worth testing whether this absence of an adverse effect of poetic deviations can be replicated for familiar versus unfamiliar poems and humouristic couplets. A positive result would imply that the Hypotheses 2a and 2b––for which we found no support in responses to unfamiliar texts––are likely to be well supported in self-selected repeated encounters with poems (which account for most readings of poetry outside the laboratory). The same holds for the many commercial ads and political slogans which feature both parallelisms and deviation: because repeated airing––and hence the creation of a familiarity effect––is a design feature of ads and slogans, their effects routinely rely on combining the powers of parallelism and deviation with those of familiarity.

The text-based measures used in this study not only need to go through further testing. In order to make them a feasible tool applicable without hundreds of hours of coding time, they need to be developed in the direction of being automatically codable with the help of natural language processing tools (e.g. [69,70]).

Psychological research has found substantial evidence for a particular salience and memorability of negative emotional experiences (e.g. [71,72]; for a transfer of these findings to art reception see [73]). In a similar vein, proverbs that are not just unusually parallelistic, but also include marked detours from ordinary language use have been shown to support higher memorability [40]. To the extent that negative detours from expectations leave stronger memory traces than positive ones, deviations may well enhance memorability in general and hence support an important goal shared by poems, political slogans, commercial ads and other texts. This assumption is also readily compatible with the predictive processing framework: deviations lead to prediction errors, and these lead to an updating of the internal world model, or, for that matter, the text model. This update may be accompanied by increased attention, which in turn may strengthen the memory trace that is involved and thereby enhance memorability.

## Data Availability

The stimuli, data and analysis scripts are available from the OSF repository: https://osf.io/dcre9 [74]. Additional material is also available in the electronic supplementary material [75].
